# Determining the Impact of Urban Vacant and Abandoned Land on Land Surface Temperatures in Socially Vulnerable Communities in Houston

**DOI:** 10.3390/cli14040078

**Published:** 2026-03-27

**Authors:** Dingding Ren, Galen Newman, Robert D. Brown, Dongying Li, Lei Zou

**Affiliations:** 1 Department of Landscape Architecture & Urban Planning, Texas A&M University, College Station, TX 77843, USA; 2 Department of Geography, Texas A&M University, College Station, TX 77843, USA

**Keywords:** land surface temperature, urban heat island, vacant land, abandoned land, social vulnerability, drone thermal imaging

## Abstract

Uneven urbanization can lead to significant quantities of vacant and abandoned land while exacerbating urban heat island (UHI) effects and simultaneously adversely affecting socioeconomically disadvantaged communities. This study examines the correlation between land surface temperature (LST) and urban vacant and abandoned land in socially vulnerable neighborhoods in Houston, TX, USA, where extreme heat can present significant environmental and public health challenges. Six critical study locations exhibiting a social vulnerability index (SVI) over 0.7 and average land surface temperature (LST) values surpassing 82 °F (27.8 °C) are analyzed through spatial analytics and drone footage. Findings indicate that vegetated vacant spaces help mitigate urban heat by decreasing land surface temperature, but abandoned structures exacerbate temperatures due to heat retention from non-permeable surfaces. Findings suggest that elevated socioeconomic vulnerability correlates with increased land surface temperature, exacerbating heat-related hazards in at-risk communities. In this six-site sample, the abandonment rate exhibited a positive correlation with the site mean land surface temperature (exploratory linear fit: +2.42 °F [0.74, 4.11]/+1.35 °C [0.41, 2.28] per +1% increase in abandonment; to be interpreted as exploratory and potentially confounded). Results provide critical insights for climate resilience planning and urban heat reduction through high-resolution thermal and geographical analysis, highlighting the impact of vacant and abandoned land on LST. Such findings endorse certain urban cooling techniques, including land reutilization and green infrastructure, to enhance environmental equality and adaptation.

## 1. Introduction

Urbanization is a rapid process that transforms land use and land cover (LULC), fundamentally modifying the natural environment. As urban areas expand, both green and open spaces are replaced by built infrastructure, leading to a reduction in permeable surfaces and a rise in impermeable materials such as asphalt, concrete, and glass. Such changes disrupt the natural balance of heat absorption and dissipation, leading to the formation of new microclimates that exacerbate urban heat retention [1–3]. Urbanization can, thus, fragment natural ecosystems, restricting the cooling effects of green spaces and influencing surface energy exchange. The growth of dense urban centers intensifies human activity and elevates energy consumption, contributing to increased land surface temperatures (LSTs), which are amplified by the urban heat island (UHI) effect [2]. Consequently, urban areas often exhibit elevated temperatures compared to rural locations, with significant variations influenced by LULC patterns [3].

The abundance of vacant and abandoned land in urban regions is increasingly concerning, often linked to economic recessions, zoning regulations, property trading, and shifting demographic trends [4]. Often referred to as underutilized areas, vacant and abandoned lands are frequently characterized by bare soil, deteriorating buildings, or unmanaged vegetation and are especially prevalent in urban environments experiencing economic and demographic decline [5]. Although many vacant lands revert to unmaintained natural vegetation, numerous places also comprise heat-retaining elements that exacerbate local temperature rises [6]. For example, abandoned structures can exacerbate urban decline by influencing thermal properties by retaining heat within deteriorated materials [7]. The environmental and social consequences of vacant and abandoned land extend beyond simply esthetics; they may impair urban cooling mechanisms, diminish climate resilience, and often present safety hazards [8].

The UHI effect is a well-documented phenomenon which results from the concentration of impermeable surfaces and anthropogenic activities in urban areas, leading to localized increases in temperature. The UHI phenomenon may result from various factors such as the replacement of vegetated areas with heat-retentive materials, the density and configuration of structures that retain heat, and limited airflow resulting from urban planning. Human-generated heat emissions from automobiles, industrial activities, and cooling systems intensify temperature variations between urban and rural regions [7]. LST is an important indicator for comprehending UHI dynamics, with remote sensing technologies such as Landsat thermal imagery and drone-based thermal imaging offering essential insights into the role of various land cover types in urban heat retention [9,10]. Although some research has examined the impact of developed regions on the UHI, the effect of vacant and abandoned land remains mostly unexplored. These areas, based on their physical characteristics, can either mitigate or exacerbate urban heating, making them a crucial factor in comprehending LST variations [7].

Extreme heat often impacts urban populations disparately, with socially vulnerable areas seeing a disproportionate impact from high temperatures [11]. These neighborhoods, often situated in areas with limited tree cover, high concentrations of undeveloped land, and substandard housing, are especially vulnerable to extreme heat. Many economically disadvantaged districts have traditionally faced exclusionary planning practices, such as redlining, which limited infrastructure investment, including green spaces and cooling services. Consequently, inhabitants in these regions often encounter increased LST and augmented hazards of heat-related illnesses, cardiovascular stress, and respiratory issues [12]. Economic constraints can further diminish access to adaptive measures such as air conditioning, exacerbating vulnerability [13].

The UHI effect is a thoroughly researched climatic phenomena, and many studies have linked land cover and land use to surface temperature patterns. Nevertheless, a more specific gap remains: the majority of remote-sensing and land-cover analyses categorize “vacant” areas as a generic land-cover class (e.g., open space/grass/bare soil). They also infrequently differentiate the condition of use of the property—especially the state of abandonment (e.g., disinvestment, building deterioration, prolonged inactivity) and its temporal dynamics. The thermal consequences of abandonment as a socio-spatial process are frequently associated primarily with vegetation cover or impermeable surfaces [6]. This study fills the aforementioned gap by explicitly operationalizing and mapping vacant land and abandoned structures, correlating this categorization with satellite-derived LST and drone thermal imagery, and ultimately investigating the relationship between land conditions and surface temperatures, using Houston, TX, USA, as a study site. To concentrate on exacerbated heat and social risk, we analyze six locations, at the census tract scale, which exhibit high social vulnerability (SVI > 0.7) and increased thermal exposure (mean LST > 82 °F/27.8 °C).

## 2. Materials and Methods

### 2.1. Study Design and Datasets

This study employed geospatial and statistical techniques to evaluate the correlation between LST and land-use dynamics, including vacant lots, abandoned structures, SVI, and land-use classifications. LST data were obtained from Landsat thermal satellite imagery, providing large-scale temperature measurements over Houston. Data on vacant and abandoned land was obtained from GIS-based land-use data, building footprint information, and LandSat data for land surface temperature computation (Table 1).

#### 2.1.1. Operational Criteria and Classification Process for Vacant, Abandoned, and Underutilized Land

Because land-status classification directly informs the study’s primary independent variables, we operationalized vacant, abandoned, and underutilized land using a rule-based workflow integrating parcel, land-use, and building-footprint datasets. Underutilized land refers to parcels identified in the land-use layer, which is directly derived from the spatial datasets. The identified underutilized properties were subsequently subdivided based on building footprint data. Vacant land is characterized as an underutilized parcel with no building footprint in the building footprint layer and lacking any apparent active structures in recent high-resolution base map data; these parcels may feature unmanaged vegetation, bare soil, or informal uses. Abandoned land is defined as an underutilized parcel including a building footprint, corresponding to parcels with inactive structures or those inconsistent with their observed use.

Classification was conducted in three phases: (1) the collection and spatial standardization of parcel boundaries, land-use categories, and building footprints; (2) the identification of underutilized parcels from the land-use layer, subsequently categorized into candidate-vacant (no footprint) and candidate-abandoned (footprint present) classes through rule-based subdivision; and (3) the evaluation of accuracy through the manual interpretation of a random validation subset for each class, utilizing high-resolution base maps. Validation was conducted by two independent reviewers who applied the coding framework to the same subset of records. Their outputs included reviewer-specific coding sheets, an initial measure of inter-rater agreement (e.g., percent agreement or Cohen’s kappa), and a reconciled master dataset produced after discrepancies were discussed and resolved by consensus. This process was appropriate because independent review helps assess whether categories are being applied consistently, while consensus discussions improve the clarity and communicability of the coding framework and strengthen the transparency and trustworthiness of the final analytic decisions [14].

Houston, the fourth-largest city in the United States, has had rapid population growth and extensive urbanization, leading to significant modifications in land use. As of the third quarter of 2025, the city’s office market had a high vacancy rate of 26.3% [15]. This urbanization has substituted natural vegetation with impermeable materials such as asphalt, concrete, and rooftops, intensifying the UHI effect [2,16].

#### 2.1.2. Landsat-Derived LST (2015/2019/2022)

This work utilized Landsat satellite images from three distinct temporal points to enhance the robustness and statistical reliability of the LST model: 27 September 2022 (Landsat 8), 5 October 2019 (Landsat 8), and 10 October 2015 (Landsat 8). The selected dates aim to reflect comparable seasonal conditions in early autumn, minimizing variations due to vegetation phenology and solar angle. The thermal infrared bands from each dataset were analyzed using ArcGIS Pro (Version 3.0.4), which employed raster computations to derive LST values. Each picture was individually calibrated and transformed into surface temperature utilizing the standardized single-channel method, which accounted for brightness and atmospheric correction parameters, as well as surface emissivity assumptions aligned with the single-channel approach; the single-channel approach is a method used to calculate land surface temperature (LST) using data from only one thermal band of a satellite (like Landsat 8 or 9). In addition, cloud-contaminated pixels were excluded before computing the tract-level mean LST.

### 2.2. Site Selection and Drone-Based Thermal Mapping

**Figure 1.**
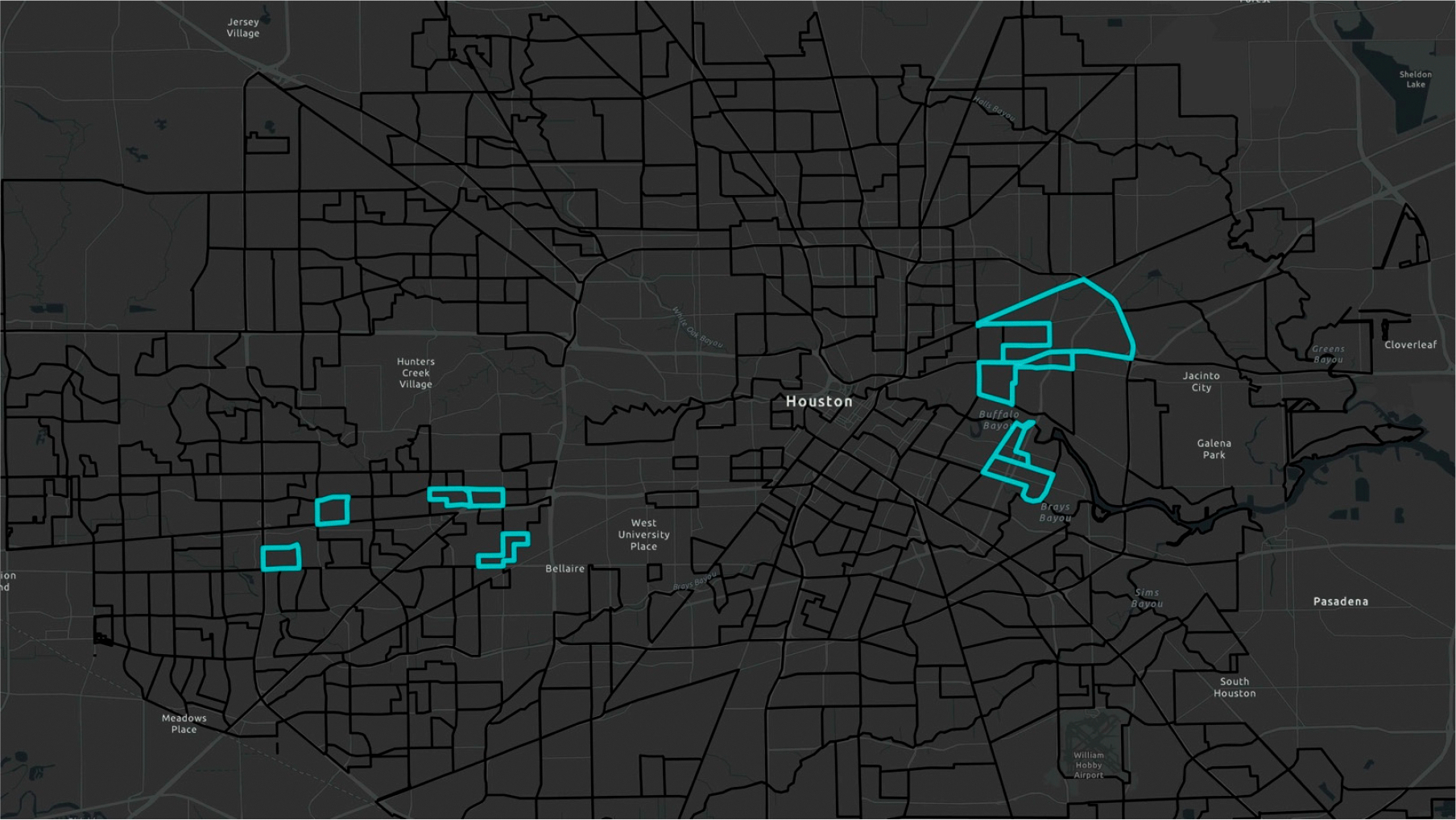
Spatial distribution of the six selected census tract study sites in Houston.

The identification of highly sensitive communities—defined in this study as census tracts with a Center for Disease Control’s social vulnerability index (CDC’s SVI) exceeding 0.7—was crucial for prioritizing areas most vulnerable to the cumulative effects of excessive heat and other environmental stresses. Areas exceeding this threshold are classified in the top 30% of social vulnerability and are acknowledged as being disproportionately impacted by climate-related hazards, such as the UHI effect. The CDC’s SVI assesses a community’s ability to prepare for, respond to, and recover from external stressors, such as natural disasters and climate change, by consolidating 15 socioeconomic variables, including income, race, age, housing status, and transportation [17,18]. Utilizing this framework to investigate the thermal effects of urban vacant and abandoned land, we identified six study sites based on integrated environmental and socioeconomic screening criteria, emphasizing areas with an SVI exceeding 0.7 and a mean LST higher than 82 °F (27.78 °C)—thresholds aligned with the upper 30% of SVI and mean LST values throughout Houston (Figure 1). The tract-level cutoffs allowed the investigation to focus on high-risk areas where increased heat exposure aligns with restricted adaptive capacity.

**Figure 2.**
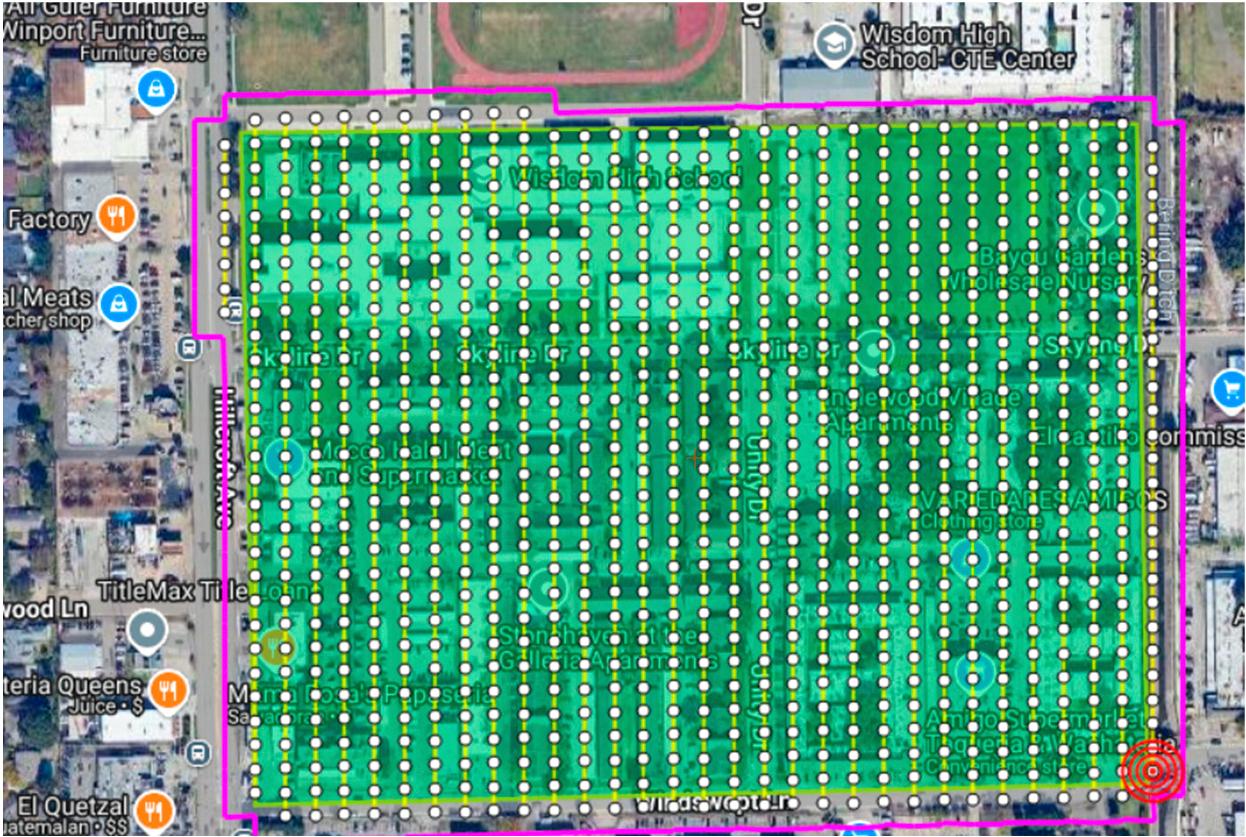
Flight plan example, site 2.

The site-level data collection incorporated the use of DJIF light Planner software to optimize the data collection process with the drone (Figure 2). This software was used to set up precise flight routes, including 50% overlap and automated photo capture every 3 s.

### 2.3. Data Processing

**Figure 3.**
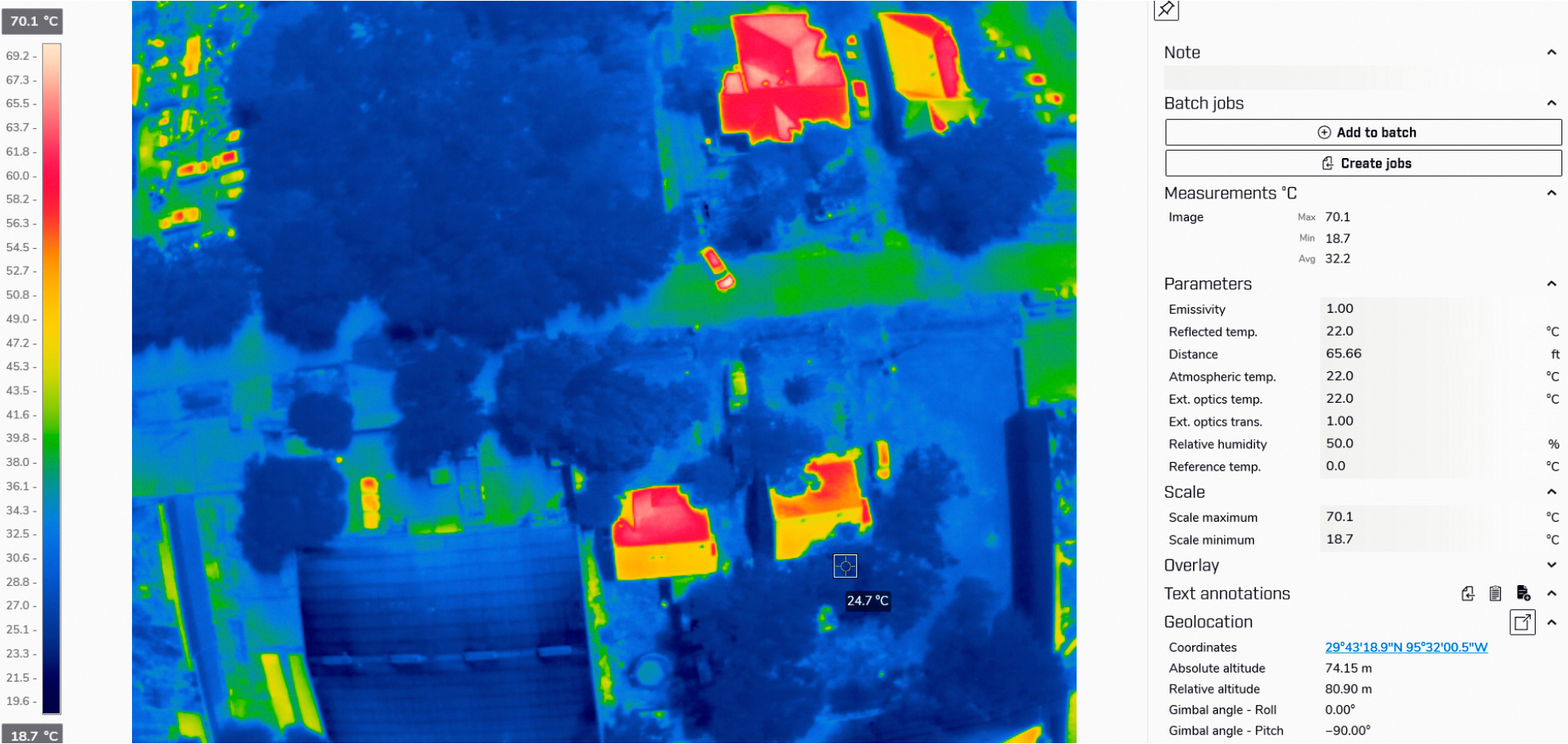
Flir Studio Pro data processing, site 1.

Before the field campaign, the flight plan was prepared as CSV data and loaded into DJI Litchi to test the mission workflow in advance, allowing the team to verify routes, settings, and overall feasibility to reduce the risk of in-field execution errors. Thermal data were collected using the DJI FLIR Zenmuse XT, a stabilized aerial thermal camera featuring a FLIR Tau 2 sensor (uncooled VOx microbolometer) with 640 × 512 or 336 × 256 resolution options. Key specifications include <50 mK thermal sensitivity, 30 Hz (NTSC) or 25 Hz (PAL) frame rates, multiple lens options (6.8–19 mm), and 3-axis gimbal stabilization [19], supporting consistent image capture during flight. After data collection, raw thermal photographs were processed in FLIR Studio Pro (Version 1.9.95) (Figure 3), which was used to convert the original files into interpretable thermal images and to extract key descriptive metrics, specifically minimum and maximum temperature values.

**Figure 4.**
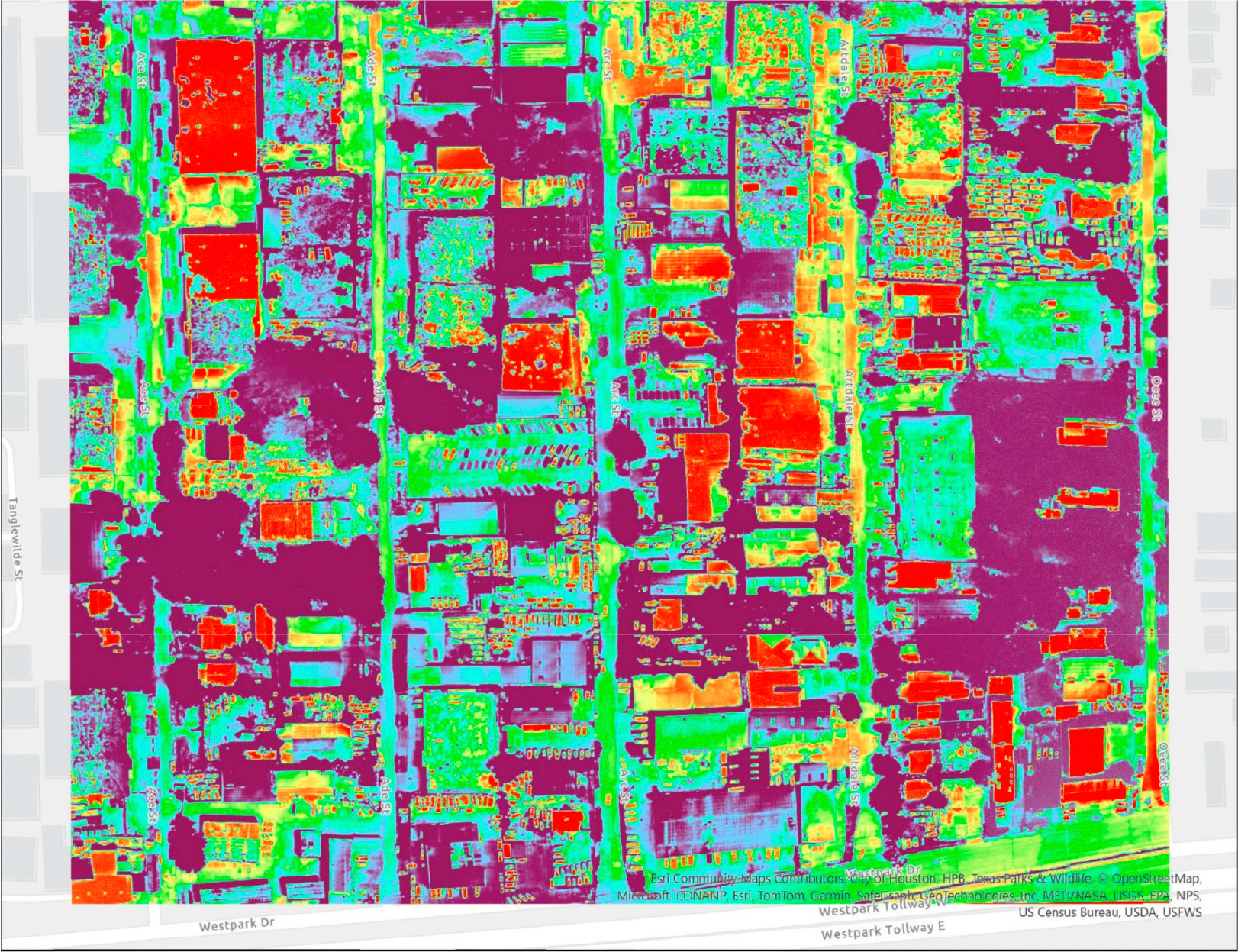
Thermal intensity map stitched, site 1.

To ensure image quality and spatial continuity across the study area, Pix4D was then employed to correct fisheye distortion and to stitch individual thermal frames into seamless thermal mosaics (Figure 4). Finally, the resulting thermal products were imported into ArcGIS Pro (Version 3.0.4) for georeferencing and rescaling, so they aligned accurately with real-world coordinates, enabling subsequent spatial analysis and integration with complementary geospatial datasets for mapping, comparison, and interpretation.

#### 2.3.1. UAV Thermal Calibration

Radiometric UAV thermal imagery was calibrated and quality controlled following published best-practice guidance for UAV thermal infrared data, which emphasizes that accurate surface temperature retrieval depends on stable camera behavior during flight and physically consistent specification of emissivity and atmospheric terms (humidity, distance, and reflected background temperature). Each thermal frame was processed using the radiometric parameters recorded in the FLIR interface to convert measured longwave radiance to temperature. For the representative scene, the parameter panel indicates emissivity (ε) = 1.00 (inspection baseline), reflected apparent temperature (T_refl) = 27.0 °C (80.6 °F), atmospheric temperature (T_atm) = 27.0 °C (80.6 °F), relative humidity = 50%, sensor-to-target distance = 65.66 m (215.4 ft), IR optics temperature = 22.0 °C (71.6 °F), and external optics transmission = 1.00 (no external lens). These inputs were applied to account for atmospheric attenuation along the viewing path (distance- and humidity-dependent), downwelling longwave radiation reflected by target surfaces (T_refl), and camera/optics self-emission (optics temperature), consistent with the principal drivers of UAV thermal infrared uncertainty described in the literature [20].

To reduce bias associated with unstable camera output early in a flight—commonly reported for uncooled microbolometer systems—frames collected during takeoff and any periods showing abrupt shifts, striping, or non-physical gradients—were excluded prior to mosaicking, and correction settings were held constant within each flight to minimize within-survey drift effects. Because emissivity is frequently the largest source of uncertainty when translating radiance to absolute surface temperature [20], the ε = 1.00 inspection default was replaced during final export with surface-class emissivity values (e.g., vegetation, asphalt, concrete, roofing, water/bare soil as applicable), improving physical realism across mixed urban materials.

To evaluate robustness to plausible emissivity uncertainty, emissivity for each surface class was perturbed by ±0.02 while holding other parameters constant (T_refl = 27.0 °C [80.6 °F], T_atm = 27.0 °C [80.6 °F], RH = 50%, distance = 65.66 m, optics terms unchanged). The perturbation produced modest shifts in absolute temperatures—most pronounced for the warmest impervious materials—but preserved the rank ordering of surface types within sites (e.g., sunlit roofs/pavement remaining warmer than shaded vegetation), indicating that relative contrasts and hotspot delineation are stable to small emissivity changes [20].

#### 2.3.2. Temperature Comparability and Validation

To contextualize and validate the environmental inputs used for radiometric correction (notably T_atm and T_refl), we compared the flight-time ambient setting (27.0 °C/80.6 °F) against Houston’s early-fall climatological normals from the National Weather Service (IAH, 1991–2020): September 27 normal high/low = 88/67 °F (31.1/19.4 °C) and October 5 normal high/low = 86/65 °F (30.0/18.3 °C), while October 10 normal high/low = 85/63 °F (29.4/17.2 °C) [21].

### 2.4. Statistical Analysis

#### 2.4.1. Exploratory Association Between Vacancy/Abandonment and Site Mean LST

Given the small number of sites (*n* = 6) and mixed land-use contexts, we treated all site-scale inferential statistics as exploratory. We fitted a simple ordinary least squares regression relating site mean LST to the site abandonment rate (%). The fitted slope in this sample was 2.42 °F per +1% abandonment (1.35 °C per +1%), with a wide 95% confidence interval [0.74, 4.11] °F ([0.41, 2.28] °C). We reported this to summarize direction and magnitude, while emphasizing that unmeasured covariates (e.g., tree canopy, parking lot, and land-use type) may confound the relationship.

#### 2.4.2. Site Classification and Variable Construction

Key land-condition variables included abandoned buildings, utilized buildings, parking lots, vacant lots, and tree patches. Operational definitions and the vacancy/abandonment identification workflow are provided in Section 2.1.1. In rare cases where a land surface category was not present within the usable thermal mosaic extent, that category was recorded as not available (NA) and excluded from the surface-type summary for that site. To avoid overclaiming, results from this planned analysis should be presented as association estimates with clearly stated assumptions and limitations, and any causal language should be avoided unless a stronger design (e.g., quasi-experimental or longitudinal) is implemented.

A representative set of six medium-sized urban sites was selected to reflect a range of land-use contexts and land-condition types (vacant lots, abandoned structures, parking lots, and tree patches). For each site, we computed the proportional area (%) of each class. These class proportions were then related to site mean LSTs derived from the drone thermal mosaics and summarized descriptively by land-use type (commercial/residential/industrial).

## 3. Results and Discussion

### 3.1. City-Scale Patterns, LST and Vulnerability

**Figure 5.**
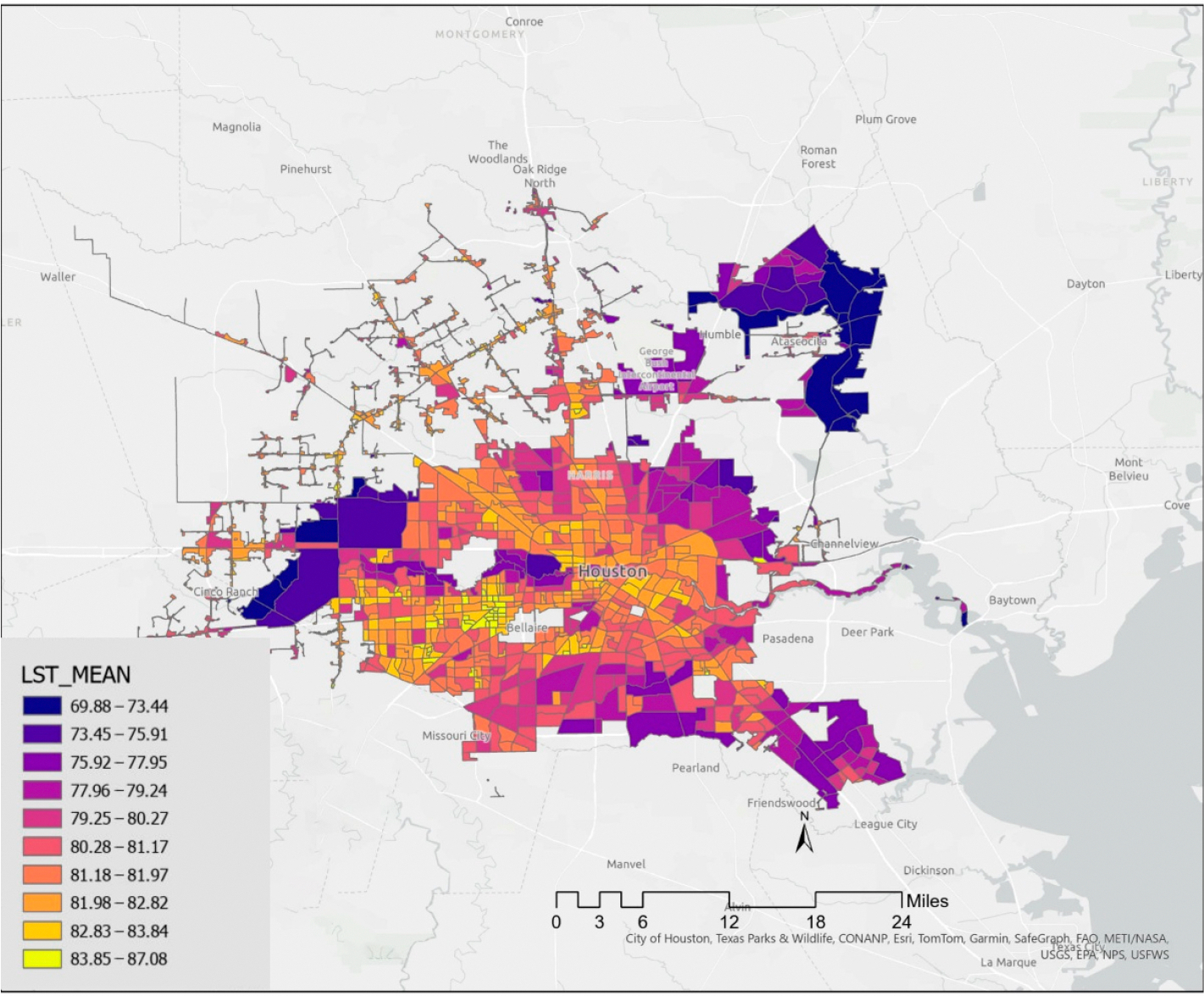
The average LST in Houston by census tract.

The average LST by census tract within the Houston area (Figure 5) shows a distinct UHI pattern. Higher temperatures, highlighted in yellow and orange at a rate of 83.85–87.08 °F (28.81–30.60 °C), are concentrated in central Houston, particularly in densely populated regions with little vegetation. Cooler places, seen in blue and purple at a temperature of 69.88–75.95 °F (21.04–24.42 °C), are in the suburbs, such as The Woodlands and Katy, where tree cover and open space are more common. The output shows disparities in surface heat that are frequently associated with socioeconomic vulnerability, highlighting the need for targeted cooling methods in historically underserved urban areas.

**Figure 6.**
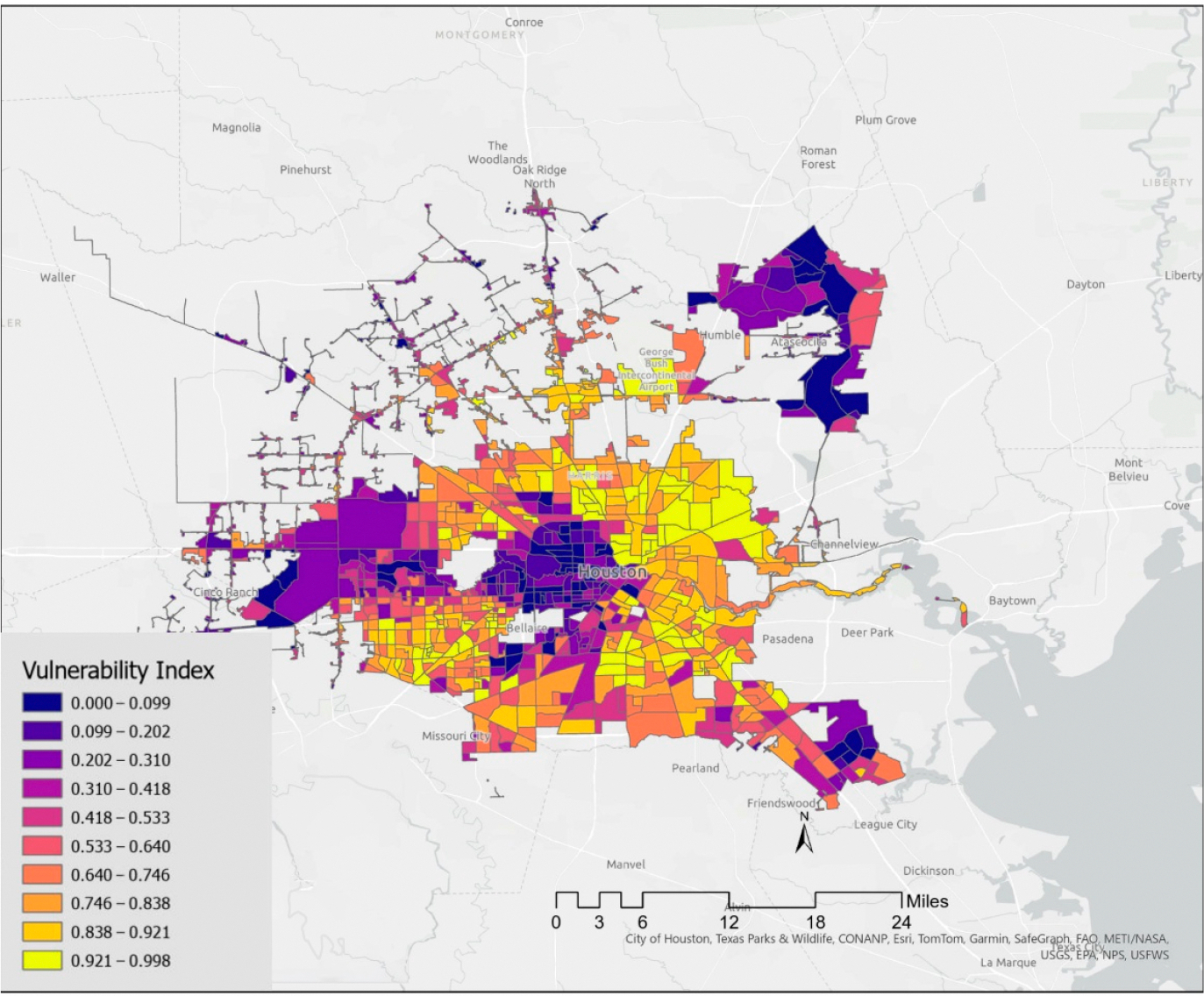
The average SVI in Houston by census tract.

The average SVI by Houston-area census tract (Figure 6) shows that higher vulnerability zones, highlighted in yellow and orange (SVI 0.748–0.998), are found in Houston’s center, southeastern, and southwestern regions, including traditionally disadvantaged neighborhoods. Lower vulnerability areas (SVI 0.000–0.202), shown in dark blue and purple, are also mostly found on the outer edges of the city and in suburban areas such as The Woodlands and Katy. The output highlights the uneven distribution of social vulnerability, highlighting the importance of focused planning and resource allocation to promote climate resilience in the most vulnerable populations.

### 3.2. Abandonment and Vacancy

**Figure 7.**
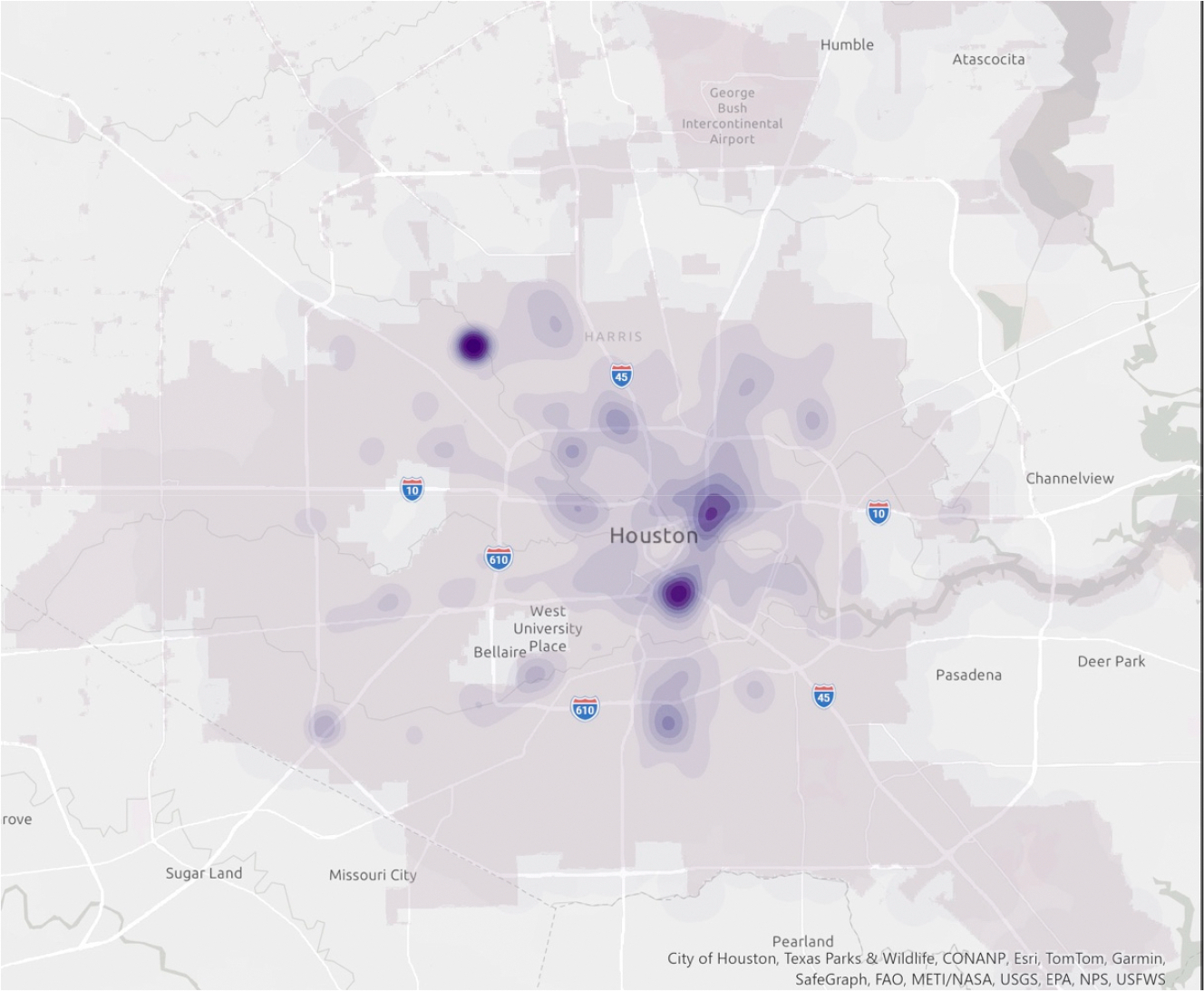
Kernel density of abandoned land in Houston (10,341 acres).

**Figure 8.**
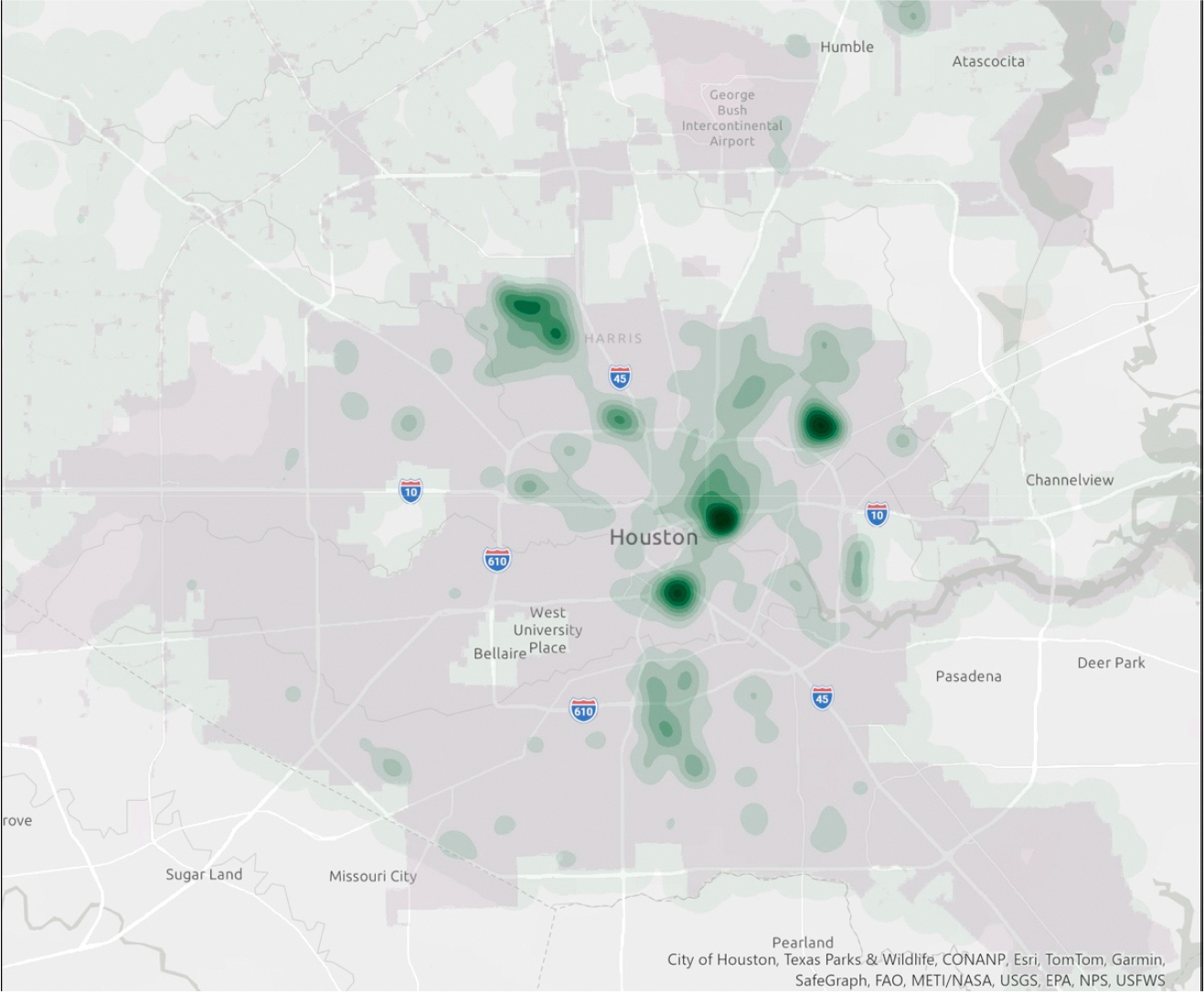
Kernel density of vacant land in Houston (45,824 acres).

The two heat maps indicate that vacant and abandoned land in Houston is distributed unevenly, exhibiting distinct geographical clustering instead of a uniform pattern throughout the city. Both maps indicate that the biggest densities are located in and around the inner city, especially near central Houston, extending towards the east, southeast, and some areas of the northwest. The maps indicate that various land conditions are closely linked to certain metropolitan areas rather than being randomly distributed (Figures 7 and 8).

A detailed comparison reveals that the vacant land map possesses a more extensive and continuous spatial footprint, featuring various clusters dispersed throughout central, eastern, southern, and northwestern Houston (Figure 8). Conversely, the abandoned land map has a more compact and concentrated distribution, resulting in fewer yet more intense hot spots, particularly in the northwest and in regions immediately south and east of downtown (Figure 7). This disparity signifies that vacant land is more extensively distributed over the city, while abandoned land is concentrated in smaller, high-density areas. Both patterns indicate regions of disinvestment and land-use shift in inner Houston, while also emphasizing that vacant and abandoned land may have specific spatial repercussions for neighborhood conditions and urban heat exposure (Figures 7 and 8).

**Figure 9.**
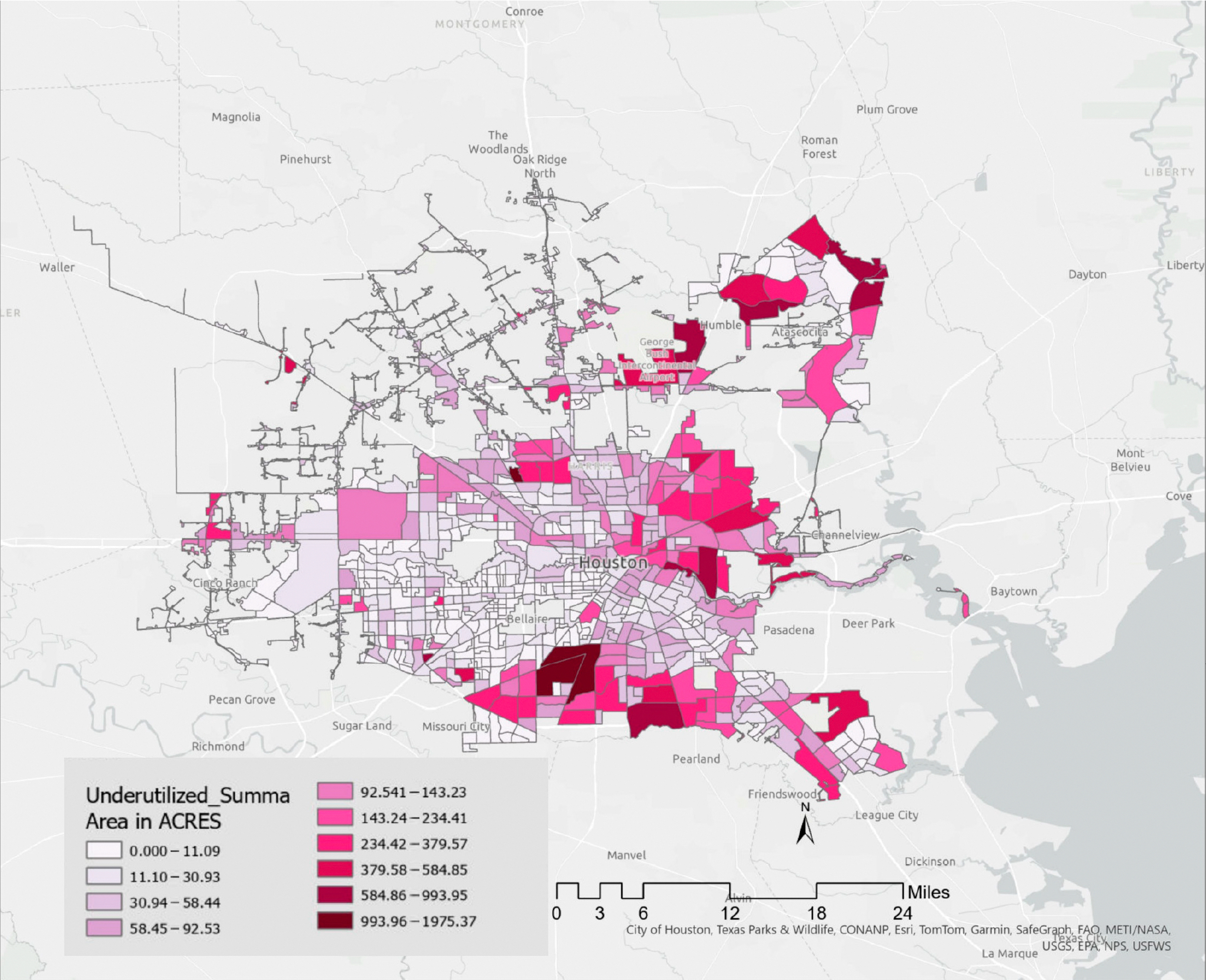
Spatial distribution of underutilized land by census tract in Houston.

The spatial distribution of underutilized land area across the Houston region reveals a markedly uneven pattern in which higher acreages cluster in specific locations, rather than occurring uniformly across the study area (Figure 9). The highest totals (darkest classes) are concentrated primarily in tracts south and southeast of central Houston, with additional high-acreage pockets along portions of the eastern corridor and in outlying northeastern/northern areas. In contrast, many tracts in central and western Houston fall into lower percentages, indicating comparatively limited underutilized land in those locations.

### 3.3. Site-Scale Thermal Dynamics from Drone Imagery

#### 3.3.1. Site Sample and Land-Use Typology

As noted, six study sites in Houston were assessed using drone-derived thermal mosaics. Sites 1–3 were categorized as commercial, sites 4–5 as residential, and site 6 as industrial. The abandonment–temperature relationships are summarized in Table 2.

#### 3.3.2. Abandonment Rate and Mean LST by Land-Use Type

**Figure 10.**
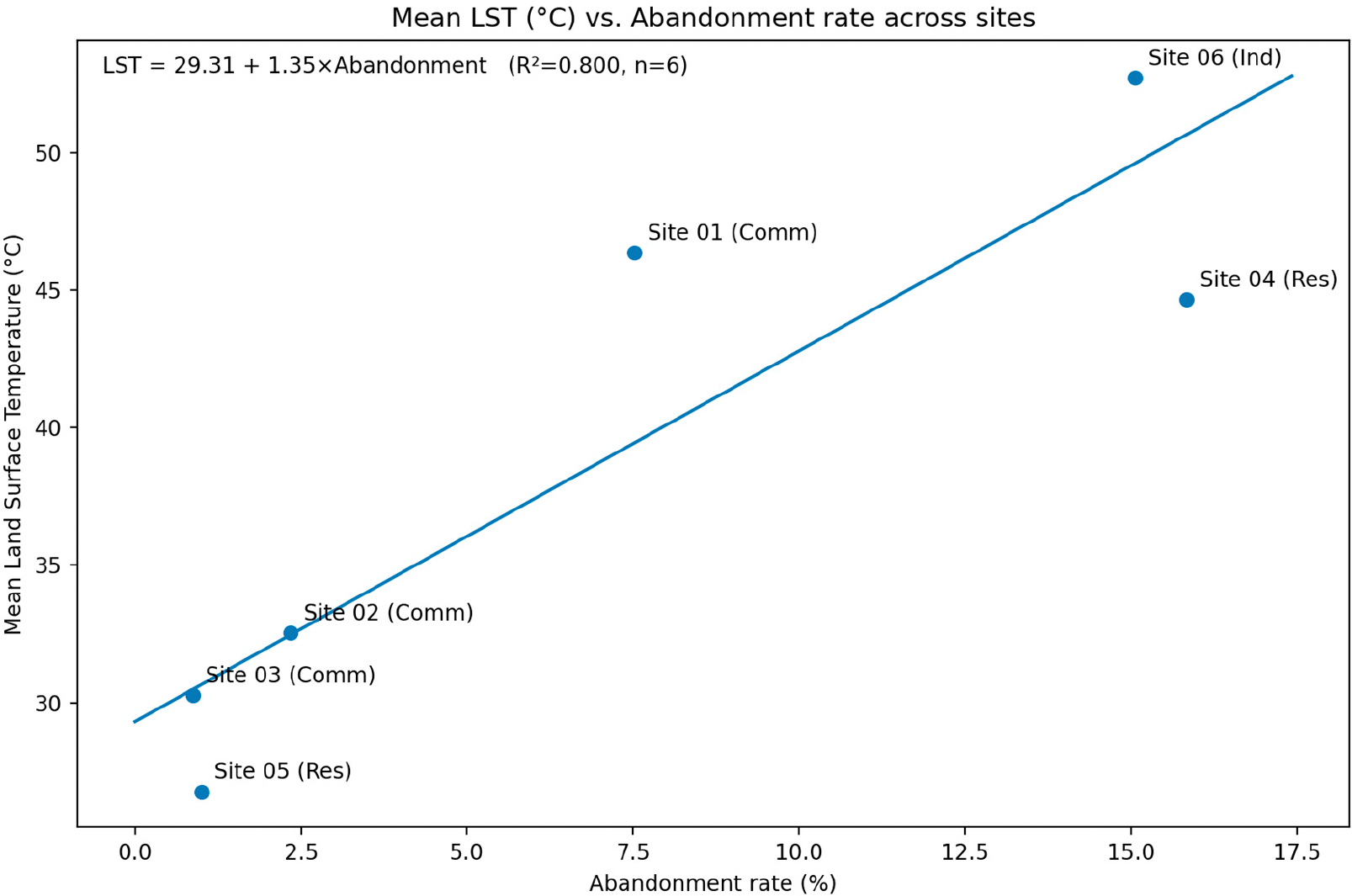
LST vs. abandonment rate by land-use type.

Across the six sites, higher abandoned rates generally align with higher site mean LSTs, though the pattern varies by land-use type. The industrial site shows both a high abandonment rate (15.062%) and the highest mean LST (126.87 °F/52.71 °C). In residential areas, the high-abandonment site (15.838%) is also relatively hot (112.39 °F/44.66 °C), while the low-abandonment residential site (0.996%) is the coolest overall (80.18 °F/26.77 °C). The three commercial sites illustrate a similar gradient: as abandonment decreases from 7.522% to 0.869%, mean LST drops from 115.43 °F (46.35 °C) to 86.50 °F (30.28 °C), suggesting that lower abandonment is associated with cooler surface conditions in this sample. Figure 10 shows that, within this six-site sample, the abandonment rate was positively associated with site mean LST (exploratory linear fit: +2.42 °F [0.74, 4.11]/+1.35 °C [0.41, 2.28] per +1% abandonment (Figure 10); interpret as exploratory and potentially confounded).

### 3.4. Land-Use Breakdown Patterns

**Figure 11.**
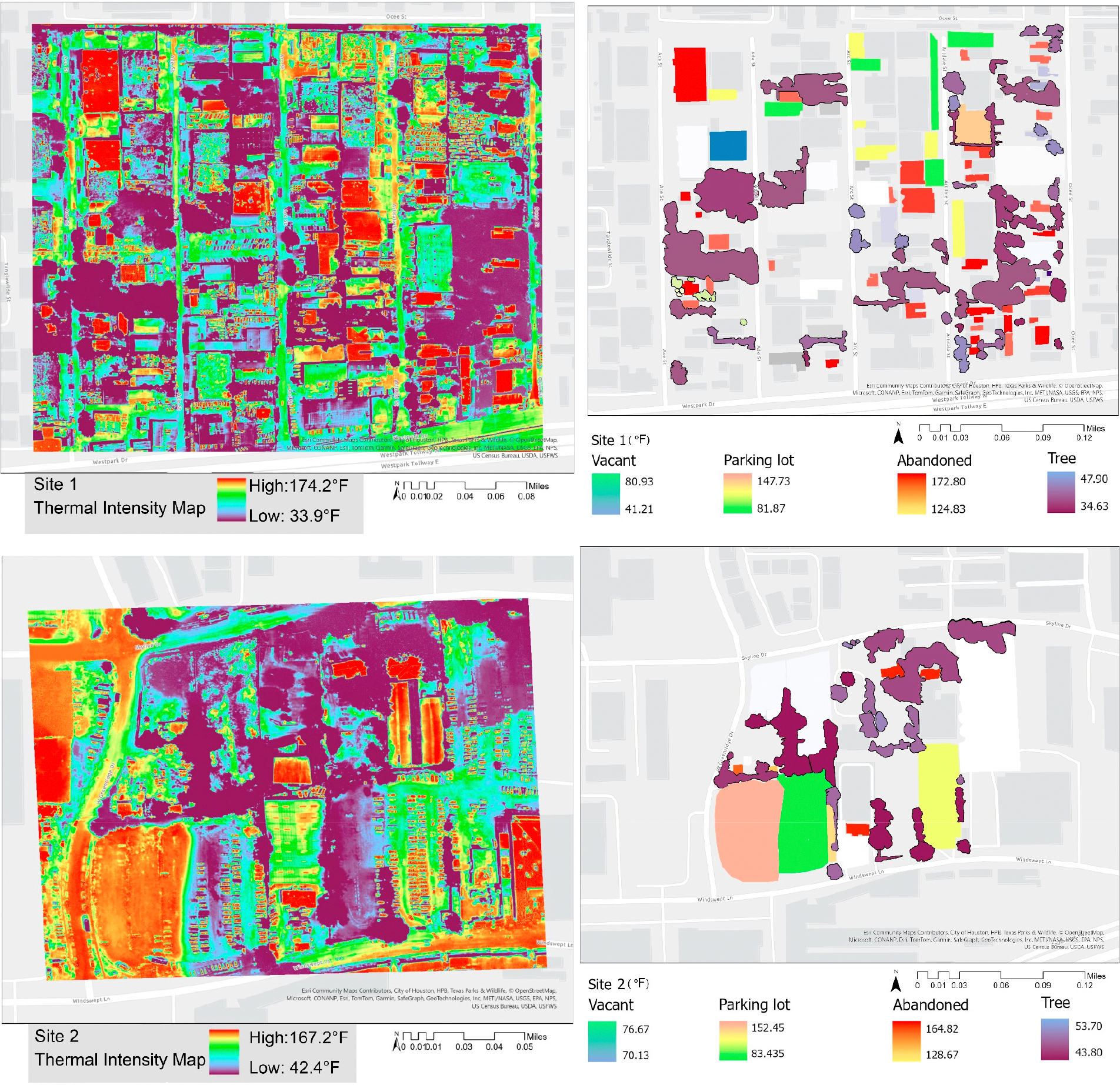

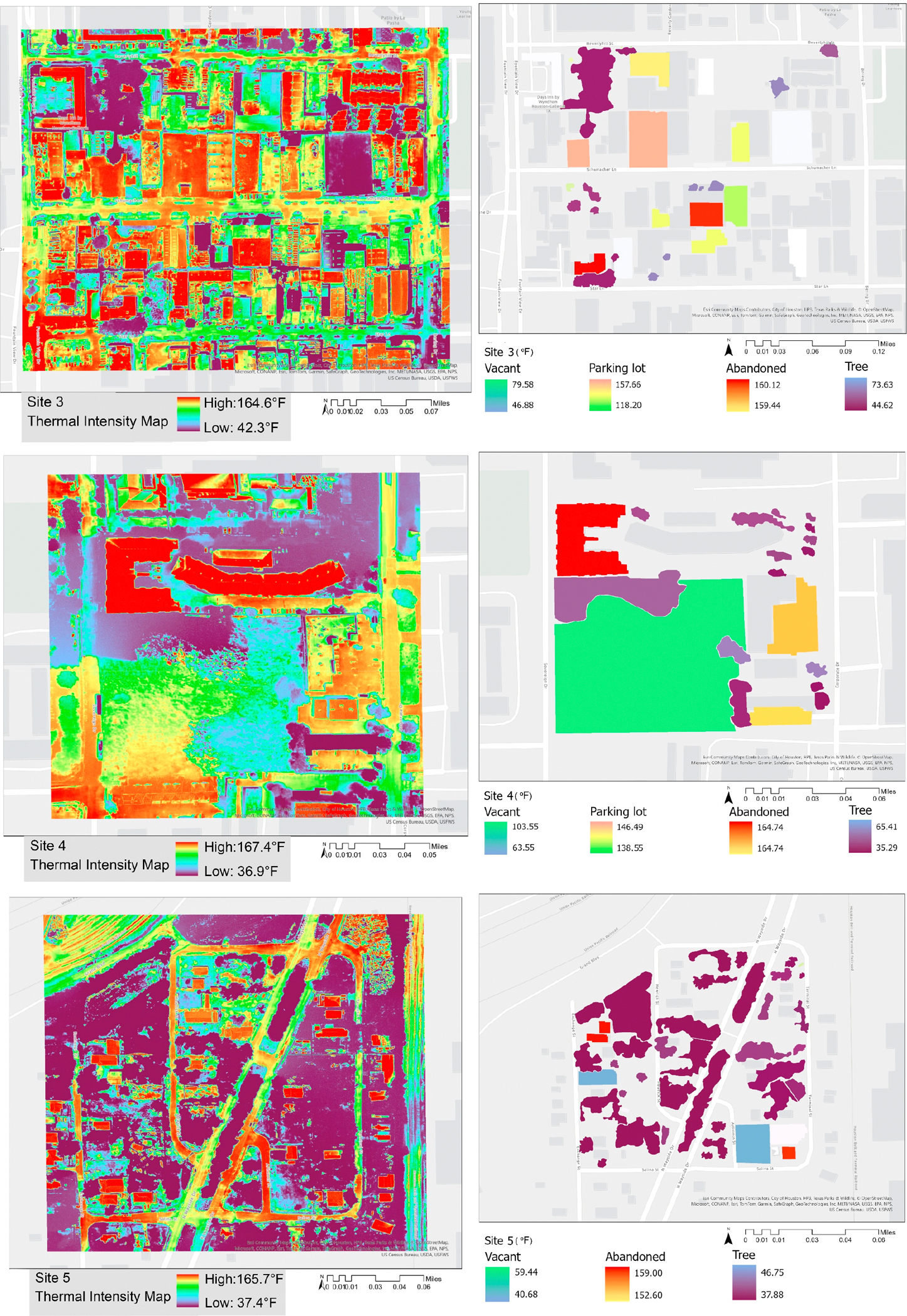

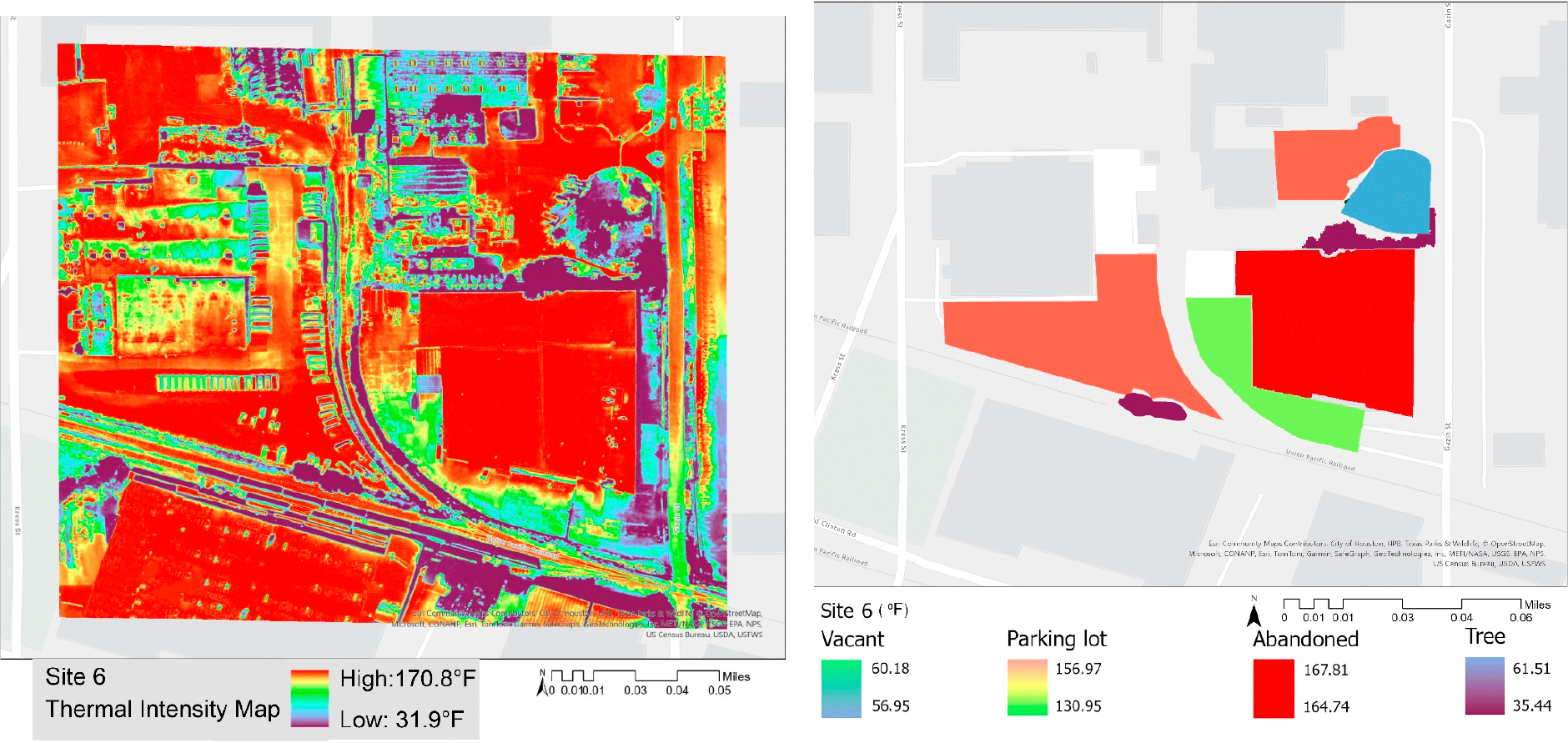
Sites 1–6 LST and land-use breakdown mean LST.

Across all sites, there was a clear and consistent thermal hierarchy by surface type with the built environment (Table 3), with abandoned structures exhibiting the highest temperatures. Abandoned buildings were the hottest surface category overall, averaging 161.28 °F (71.82 °C) across sites and ranging from 151.64 to 167.81 °F (66.47–75.45 °C). By comparison, parking lots also sustained a high LST mean of 128.99 °F (53.88 °C), ranging from 112.21 to 143.69 °F (44.56–62.05 °C), and frequently exceeded the mean of utilized buildings, which was 122.67 °F (50.37 °C), ranging from 88.99 to 154.42 °F (31.66–68.01 °C). In contrast, tree patches consistently represented the coolest condition in every site, with a mean of 53.79 °F (12.11 °C) and ranging from 51.67 to 56.11 °F (10.93–13.39 °C), while vacant land were generally cooler than built and paved surfaces (mean 68.97 °F [20.54 °C]), which ranged from 51.05 to 103.55 °F (10.58–39.75 °C). The magnitude of contrast between thermally extreme and vegetated surfaces was substantial; abandoned buildings exceeded nearby tree patches by 95.53–113.54 °F (53.08–63.08 °C) across all sites, and (where measured) parking lots exceeded tree patches by 56.11–89.42 °F (31.18–49.68 °C). This suggests that there is pronounced heat amplification associated with impervious and deteriorated built structures (Figure 11).

It is important to note that the relationship between abandoned and utilized building surface temperature was not uniform across contexts. While several sites exhibited only moderate temperature elevations in abandoned buildings relative to utilized ones—including site 2 (+26.41 °F/+14.68 °C), site 3 (+31.08 °F/+17.26 °C), and site 4 (+33.78 °F/+18.76 °C)—other locations demonstrated substantial thermal differentials, most notably site 1 (+77.21 °F/+42.90 °C) and site 6 (+60.20 °F/+33.44 °C).This spread suggests abandonment status alone does not fully determine thermal behavior. Site-specific characteristics (e.g., construction materials, roof condition, surrounding pavement, shading/trees, and adjacency to heat-generating land uses) likely also help mediate whether abandoned structure translates into extreme surface heating. Table 3 shows the LST across land types in each site. There is a data gap at site 5, where parking lot temperature is not reported, but the remaining categories still indicate a strong vegetation cooling signal (tree patch: 51.67 °F (10.93 °C)) relative to the warmer built surfaces (abandoned building: 157.41 °F [69.67 °C], utilized building: 154.42 °F [68.01 °C]).

At the site scale, mean temperature appears closely aligned with abandonment intensity. The highest abandonment rates occurred in site 4 (Residential, 15.838%) and site 6 (Industrial, 15.062%), and these sites also exhibited elevated mean temperatures. Site 6 had the highest mean at 126.87 °F (52.71 °C). Conversely, the lowest abandonment rates in commercial areas (site 3: 0.869%; site 2: 2.343%) corresponded to lower site means (86.50 °F [30.28 °C] and 90.58 °F [32.54 °C], respectively). An exploratory correlation across all six observations indicates a strong positive association between abandonment rate and mean temperature of each site (r ≈ 0.80), suggesting that higher abandonment may co-occur with thermal conditions conducive to intensified surface heating. Taken together, these results support the interpretation that structural abandonment—especially when coupled with extensive impervious cover—can amplify localized heat exposure, while even small tree patches function as strong thermal refuges within otherwise heat-prone urban fabrics (Figure 11).

These findings offer robust empirical evidence for the idea that abandonment and impermeable surface coverage substantially increase LST, whereas vegetation and active land use alleviate heat accumulation. The thermal gradient identified between abandoned infrastructure and green areas across several land-use categories indicates that strategic land management—specifically transforming vacant or abandoned sites into green spaces—can function as an effective technique for mitigating urban heat [22]. This evidence underscores the need to distinguish between land-use typologies in urban thermal evaluations and promote land reuse methods that are climate-responsive and contextually appropriate.

## 4. Conclusions

This study examined six high-risk sites in Houston, situated in socially vulnerable census tracts (SVI > 0.7) with increased thermal exposure (mean LST > 82 °F/27.8 °C). It combined spatial analysis with drone-captured thermal imagery and satellite-derived LSTs to assess the impact of varying land conditions (e.g., vacant vegetated parcels versus abandoned structures and other impervious surfaces) on local land surface temperatures. The analysis revealed that, in this six-site sample, abandonment rate was positively correlated with site-scaled mean land surface temperature (exploratory linear fit: +2.42 °F [0.74, 4.11]/+1.35 °C [0.41, 2.28] per +1% abandonment; to be interpreted as exploratory and potentially confounded). At the site level, vacant land had a significant cooling impact, presumably attributable to its vegetative cover and minimal impermeable surfaces. This pattern indicates that vacant land, when maintained in a natural or semi-natural condition, offers essential environmental advantages through passive cooling, particularly in vulnerable communities lacking formal green infrastructure. Nevertheless, these regions could enhance their cooling effect by efficient management or improved green infrastructure development.

This study’s findings emphasize vacant land management as a crucial mechanism for enhancing urban climate resilience. Abandonment, particularly in commercial and industrial zones, correlates significantly with elevated LST, indicating that rehabilitating abandoned buildings and underutilized properties may function as an economical and scalable approach to mitigate the UHI effect. Transforming vacant land into green infrastructure—such as community gardens, rewilded areas, or tree planting—can improve local thermal regulation and provide significant advantages to those most susceptible to excessive heat.

The results further endorse the integration of high-resolution thermal sensing into standard urban planning and policy-making processes. Drone-derived thermal imagery can reveal fine-scale heat variations and assist in assessing LST patterns across various land-use categories, facilitating evidence-based zoning and the strategic distribution of cooling measures [9,10]. Simultaneously, community-driven strategies for adaptive land reuse are crucial: engaging residents in the stewardship of vacant land enhances social cohesion and promotes contextually suitable cooling solutions, such as shade trees and water-wise landscaping, thereby ensuring that interventions align with local needs and enhancing vulnerable neighborhoods. Collectively, these implications indicate a multi-scale approach that incorporates scientific data, policy innovation, and community engagement to mitigate urban heat stress and foster more climate-resilient, inclusive urban environments.

Planners and researchers can more effectively mitigate the increased vulnerabilities resulting from environmental exposure and social marginalization by assessing LST at various levels. Combining macro-level patterns with micro-scale site variables enables the development of comprehensive and equitable climate adaptation plans in heat-affected urban regions. This method facilitates extensive policy formulation and tailored solutions to community requirements. By identifying specific heat hotspots and correlating them with socioeconomic data, targeted investments—such as forest restoration, reflective roofing, or park creation—can be directed to the areas of greatest need.

Urban planning policy should begin to progressively include high-resolution thermal sensing technologies into normal planning and decision-making procedures. Drone-based thermal data allows city officials to discover localized heat variations and assess LST patterns across land-use typologies [2]. Such data integration enables evidence-based zoning and the smart allocation of cooling resources. Policies that incorporate remote sensing into land-use and abandonment monitoring systems will be better positioned to handle climate concerns [10].

Community-driven initiatives for climate-adaptive land reuse are vital for effective vacant land management, since resident engagement enhances social cohesion and facilitates locally customized heat-mitigation strategies, including shade-tree installation and water-wise landscaping. Empowering neighborhoods with decision-making authority and resources promotes environmental justice and ensures that revitalization initiatives align with the experiences of those most impacted by neglect and severe heat. These participatory methods correspond with resilience planning frameworks that prioritize adaptability and environmental efficacy, highlighting the necessity for a multi-tiered urban resilience strategy that incorporates scientific thermal analysis, policy innovation, vacant land rehabilitation, and enduring community collaboration to mitigate urban heat impacts while promoting climate-resilient and socially inclusive urban settings.

## 5. Limitations

This study has several limitations related to data resolution, temporal coverage, logistics, and generalizability. Landsat-8 thermal imagery provides broad coverage but its 30–100 m resolution and 16-day revisit cycle limit the detection of fine-scale heat patterns and short-term temperature variability. Drone thermal imaging offers higher spatial detail but was collected at limited time points and not frequently enough to capture full temporal dynamics. Drone data collection was also constrained by regulatory requirements, restricted airspace, weather sensitivity, and short battery life, increasing operational complexity. Socioeconomic vulnerability was measured using the CDC’s SVI, which is updated infrequently and typically aggregated at the census-tract level, potentially masking rapid demographic change and within-neighborhood differences. LST estimates can be influenced by atmospheric conditions and surface emissivity differences across materials and integrating Landsat and drone products required substantial normalization and georeferencing that may introduce alignment errors. Findings may not be fully generalizable beyond Houston due to its distinctive climate, development patterns, and governance context. Finally, computational constraints and the absence of ground-based sensors limited processing efficiency and reduced opportunities for in situ validation. In addition, the site sample was purposively selected from high-vulnerability tracts (SVI > 0.7), which introduces selection bias in that the observed abandonment–temperature association may differ in lower-vulnerability or higher-investment neighborhoods with different building conditions, tree canopy, and maintenance regimes. Therefore, the findings should be interpreted as most applicable to high-risk contexts. Future work should test whether the same relationships hold across a broader SVI gradient and with larger samples. The final limitation of this study is that the site-level statistical analysis is observational and cross-sectional, so the reported relationships should be interpreted as associations rather than causal effects. Because the six sites were purposively selected from high-risk tracts and the analysis relies on a small sample, the estimated abandonment–LST relationship may be confounded by unmeasured or imperfectly measured covariates (e.g., tree canopy, impervious fraction, building materials/roof condition, shading, and adjacent land uses) and may not generalize to lower-vulnerability or higher-investment neighborhoods.

## Figures and Tables

**Table 1.** Secondary data.

Data	Company	City, State, Country	Access link	Software/Version
Landsat 8 sensing data	U.S. Geological Survey (USGS)	Reston, VA, USA	https://www.usgs.gov/landsat-missions/landsat-data-access (accessed on 28 January 2026)	ArcGIS Pro Version 3.0.4
Building footprints	Koordinates (data access platform); source data from City of Houston	Auckland, New Zealand; Houston, TX, USA	https://koordinates.com/layer/12890-houston-texas-building-footprints/ (accessed on 28 January 2026)	ArcGIS Pro Version 3.0.4
Land use/land cover data	Houston-Galveston Area Council (H-GAC)	Houston, TX, USA	https://www.h-gac.com/land-use-and-land-cover-data (accessed on 28 January 2026)	ArcGIS Pro Version 3.0.4
Social Vulnerability Index (SVI)	Agency for Toxic Substances and Disease Registry (ATSDR), Centers for Disease Control and Prevention (CDC)	Atlanta, GA, USA	https://www.atsdr.cdc.gov/place-health/php/svi/index.html (accessed on 28 January 2026)	ArcGIS Pro Version 3.0.4
Parcel data	Regrid (Loveland Technologies, LLC)	Detroit, MI, USA	https://app.regrid.com/us/tx/harris/houston# (accessed on 28 January 2026)	ArcGIS Pro Version 3.0.4

**Table 2.** Relationship between land use, abandoned rate, and site mean surface temperature across study sites.

Site	Land Use	Abandoned Rate (%)	Site Mean
Site 01	Commercial	7.522	115.43 °F (46.35 °C)
Site 02	Commercial	2.343	90.58 °F (32.54 °C)
Site 03	Commercial	0.869	86.50 °F (30.28 °C)
Site 04	Residential	15.838	112.39 °F (44.66 °C)
Site 05	Residential	0.996	80.18 °F (26.77 °C)
Site 06	Industrial	15.062	126.87 °F (52.71 °C)

**Table 3.** Abandonment rate, site mean LST, and land-use breakdown mean LST. Note: the parking lot surface temperature at site 5 is reported as NA because no distinct parking lot surface was captured within the site.

Site	Land Use	Abandoned Rate (%)	Site Mean	Abandoned Building	Utilized Building	Vacant Land/Lot	Parking Lot	Tree Patch
Site 1	Commercial	7.522	115.43 °F 46.35 °C	166.20 °F 74.56 °C	88.99 °F 31.66 °C	62.40 °F 16.89 °C	112.21 °F 44.56 °C	53.47 °F 11.93 °C
Site 2	Commercial	2.343	90.58 °F 32.54 °C	151.64 °F 66.47 °C	125.23 °F 51.79 °C	73.40 °F 23.00 °C	112.22 °F 44.57 °C	56.11 °F 13.39 °C
Site 3	Commercial	0.869	86.50 °F 30.28 °C	159.88 °F 71.04 °C	128.80 °F 53.78 °C	63.23 °F 17.35 °C	134.25 °F 56.81 °C	51.91 °F 11.06 °C
Site 4	Residential	15.838	112.39 °F 44.66 °C	164.74 °F 73.74 °C	130.96 °F 54.98 °C	103.55 °F 39.75 °C	142.57 °F 61.43 °C	55.32 °F 12.96 °C
Site 5	Residential	0.996	80.18 °F 26.77 °C	157.41 °F 69.67 °C	154.42 °F 68.01 °C	51.05 °F 10.58 °C	NA	51.67 °F 10.93 °C
Site 6	Industrial	15.062	126.87 °F 52.71 °C	167.81 °F 75.45 °C	107.61 °F 42.01 °C	60.18 °F 15.66 °C	143.69 °F 62.05 °C	54.27 °F 12.37 °C

## Data Availability

Data may not be available publicly due to privacy concern.

## Abbreviations

The following abbreviations are used in this manuscript:

LST: Land surface temperature
UHI: Urban heat island
SVI: Social vulnerability index
LULC: Land use and land cover
